# Supplementary material for: P-21 Kinase 1 or 4 Knockout Stimulated Anti-Tumour Immunity Against Pancreatic Cancer by Enhancing Vascular Normalisation
Source: Int J Mol Sci. 2025 Aug 28;26(17):8357. doi: 10.3390/ijms26178357 (PMC12427695; doi:10.3390/ijms26178357)
Supplement: Supplementary file 1 [file ijms-26-08357-s001.zip › ijms-3831197-supplementary.pdf]

Supplementary Materials

Supplementary Figures and Figure Legend

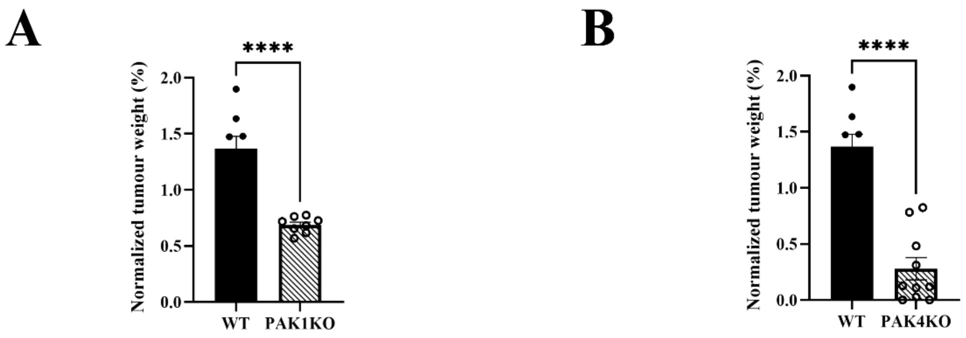

Figure S1. Knockout of PAK1 or PAK4 suppresses pancreatic tumour growth. (A) Comparison of normalised tumour weight between WT and PAK1KO groups, indicating that PAK1KO significantly suppressed tumour growth by reducing normalised tumour weight. (B) Normalised tumour weight in WT and PAK4KO groups, demonstrating that PAK4KO significantly suppressed tumour growth by reducing normalised tumour weight. ****P<0.0001.

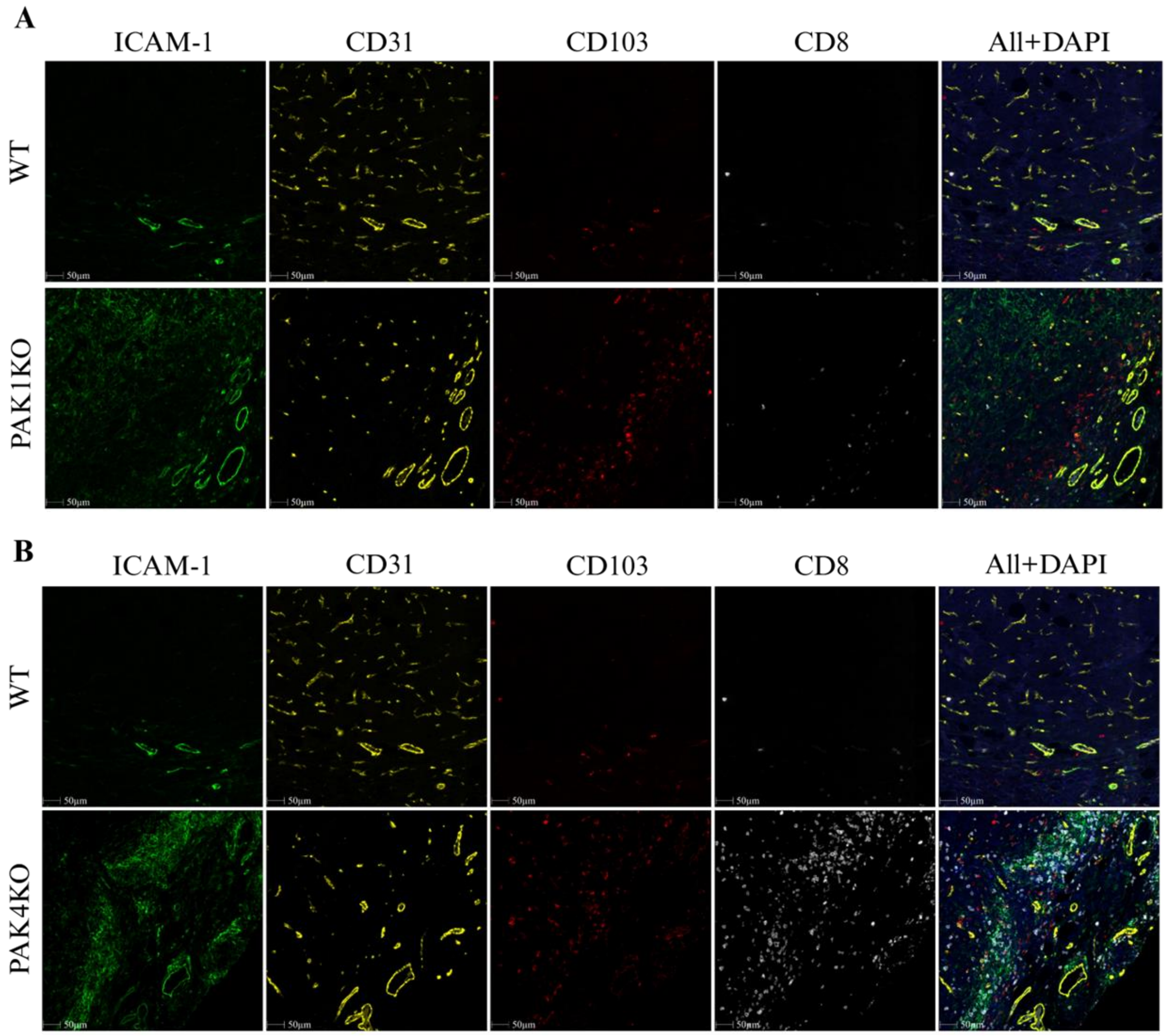

Figure S2. PAK1 and PAK4 knockout increases leukocyte trans-endothelial migration via ICAM-1 upregulation in the tumour microenvironment. Multiplex immunohistochemistry of PAK1KO (A) and PAK4KO (B) tumours revealed increased ICAM-1 expression (green) alongside markers of endothelial cells (CD31, yellow), dendritic cells (CD103, red), and T-cells (CD8, white). Representative panels showed individual marker expression and merged images (including nuclear staining (DAPI, blue)), illustrating the spatial relationship between ICAM-1^{+} cells with either vessels or infiltrating immune cell populations. These findings support a role for PAK1 and PAK4 in reducing immune cell infiltration by modulation of ICAM-1 expression.

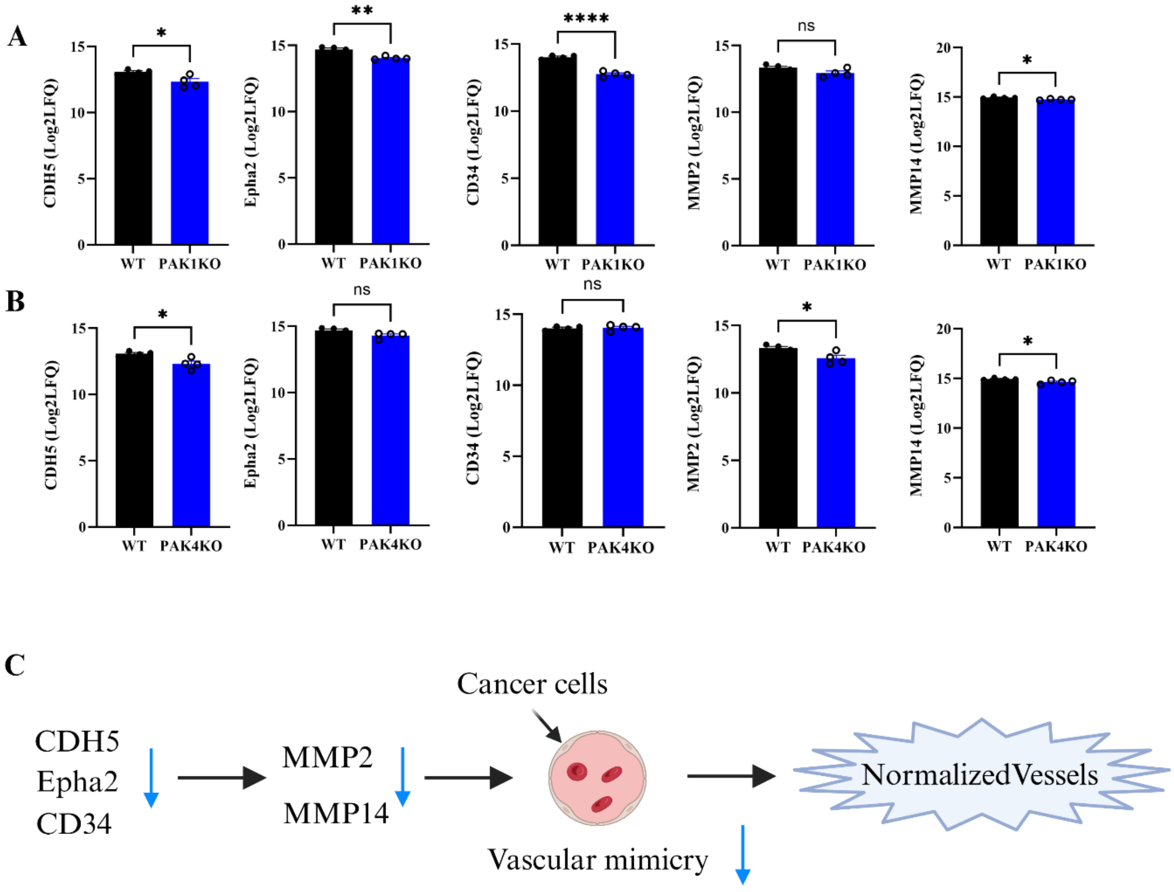

Figure S3. Proteomic evidence of reduced vascular mimicry pathways in PAK-knockout tumours. Proteomic analysis revealed significant downregulation of CDH5, EPHA2, CD34, and MMP14 in PAK1KO tumours compared with wild-type (WT) controls (A), whereas CDH5, MMP2, and MMP14 were significantly reduced in PAK4KO tumours (B). Schematic representation of the proposed mechanism, illustrating how the downregulation of CDH5, EPHA2, and CD34 suppresses the MMP2/MMP14 axis, resulting in diminished vascular mimicry in cancer cells and contributing to vessel normalisation (C). *P<0.05, **P<0.01, ****P<0.0001, ns: not significant.

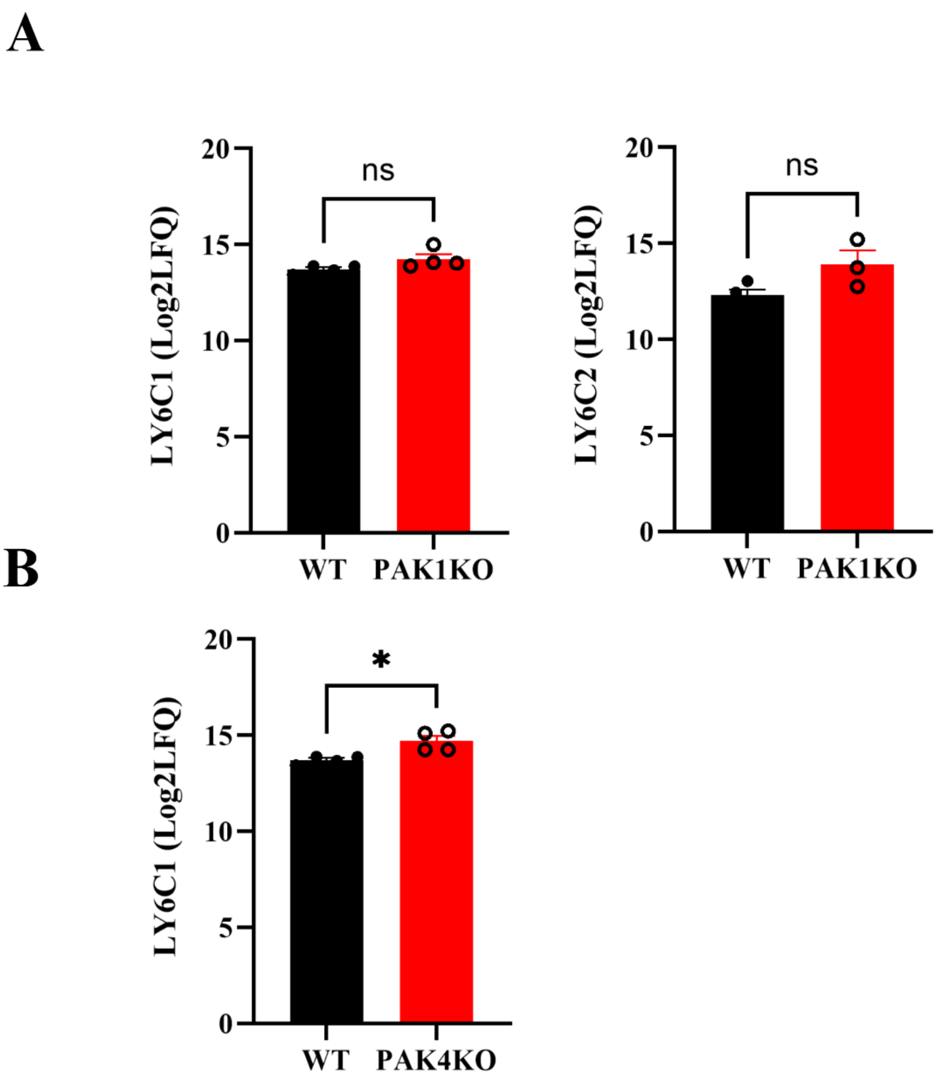

Figure S4. Proteomic analysis of LY6C expression in PAK-knockout tumours. (A) Both LY6C1 and LY6C2 levels were not changed in PAK1KO tumours compared with wild-type controls. (B) A significant upregulation of LY6C1 was observed in PAK4KO tumours. *P<0.05, ns: not significant.

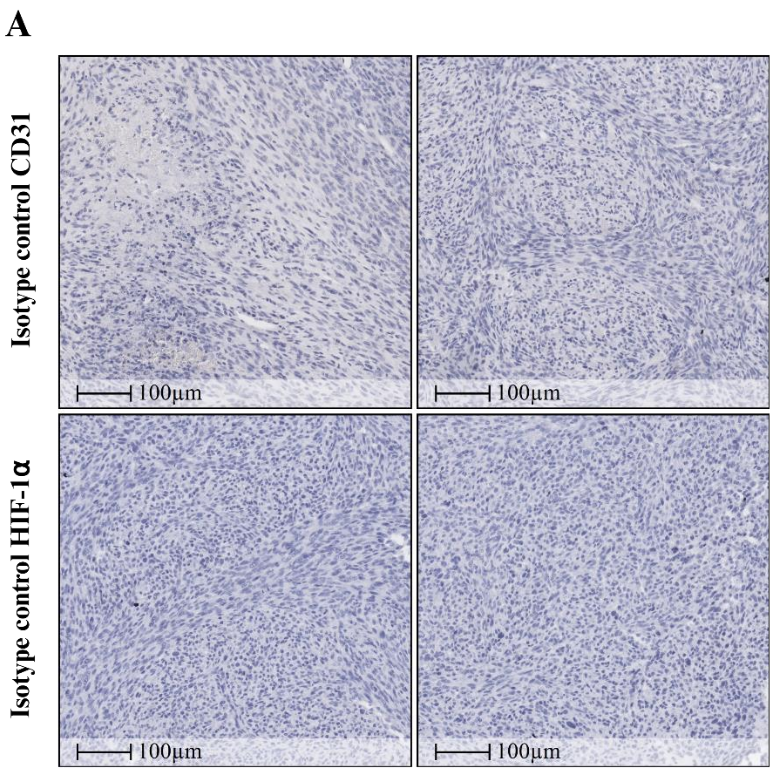

Figure S5. Isotype control validation of antibody specificity. Representative isotype control staining confirmed the absence of non-specific signal. The upper panel shows rabbit IgG control for CD31, and the lower panel shows rabbit IgG control for HIF-1α, both demonstrating no detectable staining**. Top panel was from the wild-type tumour tissue. The bottom panel was from the PAK4KO tumour tissue.**

Supplementary Tables

Table S1. Buffers used in methods.

Buffers	Content
Tris-EDTA buffer	10 mM Tris base, 1 mM EDTA solution, 0.05% Tween 20, PH 9.0
TBS-T	20 mM Tris-HCl, 137 mM NaCl, 0.1% Tween 20, pH 7.6
2x loading buffer	125mM Tris, 20% glycerol, 4% SDS, 2.5% β-mercaptoethanol, Ph 6.8
Ripa buffer	25mM Tris HCL, 150mM NaCl, 1% Triton X-100, 1% Na deoxycholate, 0.5% SDS, 1mM EGTA, PH 8

Table S2. Primary antibodies for immunohistochemistry. N/A: not applicable.

Protein target	Dilution	Cat. number	Company	Clone name
CD31	1:1500	77699S	Cell Signaling & Technology	D8V9E

CD34	1:6000	ab81289	Abcam	EP373Y
HIF-1α	1:1000	ab179483	Abcam	EPR16897
CD3	1:4000	ab5690	Abcam	N/A
CD4	1:16000	ab288724	Abcam	RM1013
CD8	1:1000	ab217344	Abcam	EPR21769
CD103	1:8000	ab224202	Abcam	EPR22590-27
CD11c	1:2000	97585S	Cell Signaling & Technology	D1V9Y
CD11b	1:64000	ab133357	Abcam	EPR1344
CD86	1:2000	19589	Cell Signaling & Technology	E5W6H
CD40	1:1000	86165S	Cell Signaling & Technology	E2Z7J
MHC-I	1:4000	76828	Cell Signaling & Technology	E8E7N
MHC-II	1:32000	PA5-116876	Invitrogen	N/A
ICAM-1	1:32000	ab179707	Abcam	N/A
VCAM-1	1:10000	ab134047	Abcam	EPR5047
Normal rabbit IgG	---	sc-3888	Santa Cruz Biotechnology	N/A

Table S3. Primary antibodies for immunofluorescent staining. N/A: not applicable.

Protein target	Dilution	Cat. number	Company	Clone name
CD31	1:1000	77699S	Cell Signaling & Technology	D8V9E
NG2	1:500	AB5320	Merck	N/A
α-SMA	1:5000	14395-1-AP	Proteintech	N/A

Table S4. Primary antibodies for multiplex immunohistochemistry. N/A: not applicable.

Protein target	Dilution	Cat. number	Company	Clone name
CD103	1:4000	ab224202	Abcam	EPR22590-27
CD8	1:1000	ab217344	Abcam	EPR21769
CD31	1:1500	77699S	Cell Signaling & Technology	D8V9E
ICAM-1	1:32000	ab179707	Abcam	N/A

Table S5. Primary antibodies for Western blot. N/A: not applicable.

Protein target	Dilution	Cat. number	Company	Clone name
MHC-I	1:1000	76828	Cell Signaling & Technology	E8E7N
MHC-II	1:1000	PA5-116876	Invitrogen	N/A

ICAM-1	1:2000	10020-1-AP	Proteintech	N/A
VCAM-1	1:2000	ab134047	Proteintech	EPR5047
PAK1	1:2000	2602	Cell Signaling & Technology	N/A
PAK4	1:2000	14685-1-AP	Proteintech	N/A
GAPDH	1:10000	2118	Cell Signaling & Technology	14C10
